# Prevalence and Correlates of Physical Activity Among Children and Adolescents: A Cross-Sectional Population-Based Study of a Rural City in Japan

**DOI:** 10.2188/jea.JE20190047

**Published:** 2020-09-05

**Authors:** Takafumi Abe, Jun Kitayuguchi, Shinpei Okada, Kenta Okuyama, Tatsunosuke Gomi, Masamitsu Kamada, Kenji Ueta, Toru Nabika, Chiaki Tanaka

**Affiliations:** 1 Center for Community-Based Healthcare Research and Education (CoHRE), Organization for Research and Academic Information, Shimane University, Shimane, Japan; 2 Physical Education and Medicine Research Center UNNAN, Shimane, Japan; 3 Physical Education and Medicine Research Foundation, Nagano, Japan; 4 Department of Health and Social Behavior, School of Public Health, Graduate School of Medicine, The University of Tokyo, Tokyo, Japan; 5 Department of Sport and Health Science, Ritsumeikan University, Shiga, Japan; 6 Department of Functional Pathology, Faculty of Medicine, Shimane University, Shimane, Japan; 7 College of Health and Welfare, J. F. Oberlin University, Tokyo, Japan

**Keywords:** exercise, school, psychosocial factor, population density, descriptive epidemiology

## Abstract

**Background:**

Although moderate-to-vigorous physical activity (MVPA) has multiple health benefits, current participation in recommended MVPA level and its determinants among Japanese children and adolescents remain unclear. Therefore, this cross-sectional study investigated the prevalence of meeting recommended MVPA level and its correlates among Japanese children and adolescents.

**Methods:**

Using the Japanese version of the World Health Organization (WHO) Health Behaviour in School-aged Children survey questionnaire, we confirmed the prevalence of meeting recommended MVPA level in all primary schools (PS) and junior high schools (JHS) in Unnan City, Japan. We evaluated its association with school grade, gender, body weight status, screen time, consumption of breakfast, physical activity (PA) preference, and population density using Poisson regression.

**Results:**

We found that 20.1% of the 1,794 students (9–15 years old) met the WHO recommendation. Meeting recommended MVPA level was significantly associated with being in the sixth grade of PS (prevalence ratio [PR] 0.57; 95% confidence interval [CI], 0.39–0.84) and first (PR 1.52; 95% CI, 1.16–1.99), second (PR 1.45; 95% CI, 1.10–1.90), and third grade of JHS (PR 0.40; 95% CI, 0.26–0.62) (vs fourth grade of PS); being a boy (PR 1.33; 95% CI, 1.12–1.59) (vs girl); liking PA (PR 3.72; 95% CI, 2.22–6.22) (vs dislike); and belonging to a medium-population-density (PR 0.73; 95% CI, 0.61–0.88) or low-population-density area (PR 0.67; 95% CI, 0.48–0.94) (vs high-population-density area).

**Conclusions:**

About 20% of Japanese children and adolescents engaged in the recommended MVPA level. MVPA was associated with grade, gender, preference for PA, and population density.

## INTRODUCTION

Regular physical activity (PA) is important for physical and mental health among children and adolescents.^1^^,^^2^ Childhood PA patterns are indicative of PA and health status in adulthood.^3^^,^^4^ Therefore, enhancing PA among children and adolescents is exceedingly important to promote public health. The World Health Organization (WHO) recommends that children and adolescents aged 5–17 years engage in at least 60 minutes of moderate-to-vigorous PA (MVPA) daily to ensure good health and fitness.^5^ However, according to the WHO Global School-based Student Health Survey (GSHS), globally, approximately 81% of adolescents aged 11–17 years do not meet this recommendation.^6^ A survey conducted by the Patient-Centered Assessment and Counselling for Exercise Plus Nutrition (PACE+) questionnaire in seven ASEAN countries showed that only 19.6% of schoolchildren aged 13–15 years engaged in more than 60 min/day of MVPA for more than 5 days/week.^7^ Therefore, physical inactivity is a common childhood health problem.

Currently, there are no representative data on MVPA for children and adolescents in Japan.^8^^,^^9^ A cross-sectional study was conducted in the Tokyo metropolitan area on population-level step counts among 15,471 students aged 6–18 years.^10^ Another study provided follow-up data on PA, which was assessed using a newly developed questionnaire^11^ and showed that 74.7% of boys and 55.2% of girls from a sample of 657 children aged 9–15 years met the PA guideline (PA level in this study was defined as ≥7 hours/week) recommended by the Japan Sports Association. However, these studies did not assess the WHO-recommended MVPA level (henceforth, recommended MVPA level). Therefore, the prevalence of meeting the MVPA recommendation remains unclear at the population level in Japan.

Previous reviews have identified a number of correlates of MVPA among children and adolescents, including demographic (age and gender), biological (health status and weight status), psychosocial (PA preference and self-efficacy), behavioral (previous PA and smoking), and environmental variables (population density and accessibility).^12^^–^^17^ The ecological model offers a useful means of structuring research on these correlates, as it places emphasis on combinations of individual and environmental factors.^15^^,^^18^ However, to the best of our knowledge, no study has yet examined the determinants of meeting recommended MVPA level using the ecological model in a community setting in Japan.

Thus, the primary objective of this cross-sectional study was to determine the prevalence of meeting the recommended MVPA level among Japanese children and adolescents. The secondary objective was to assess the relationships between this prevalence and numerous correlates.

## METHODS

### Participants

The study area was Unnan City, a rural region (area 553.4 km^2^) with no private schools in Shimane Prefecture, western Japan, with a population of 39,032; 1,932 children and adolescents aged 9–14 years lived in this city as of April 2017. Data were obtained from the health behavior surveys investigating students’ lifestyles conducted by the school nursing department of the Unnan City Board of Education in the 2017 fall academic term. The school nursing department sent a letter to parents/guardians explaining ethical considerations and requesting their participation as a volunteer. The parents/guardians were free to ask questions or refuse participation. After the survey, the school nursing department approved the secondary use of these data for research. The researchers were provided data stripped of personal identifiers. This process was handled by the school nursing department, and the researchers were not involved. We disclosed information about our study for participants on the Shimane University website. The study protocol was approved by the Ethics Committee of Shimane University (#2856).

**Figure 1.**
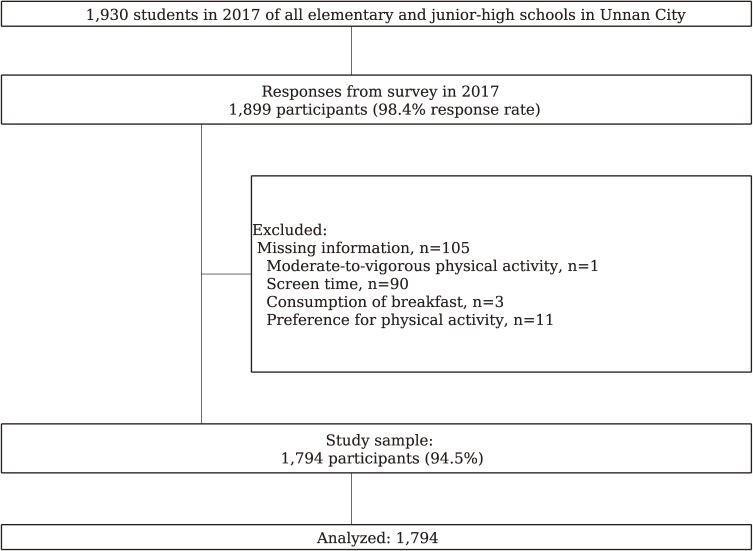
Flowchart of the study

Data were collected from health behavior surveys conducted in all public primary schools (PS; 15 schools; 950 students aged 9–12 years, grades 4–6) and junior high schools (JHS; 7 schools; 980 students aged 12–15, grades 1–3) in November 2017. Overall, 1,930 children and adolescents participated in the surveys. We excluded the data of participants who did not respond to any of the items (*n* = 31) and 105 participants with missing data (MVPA, *n* = 1; screen time [ST], *n* = 90; consumption of breakfast, *n* = 3; preference for PA, *n* = 11). When grouped according to non-missing or missing data, gender was not significantly different (*P* = 0.62), whereas school grade was significantly different (*P* = 0.045). These results are not shown. We thus conducted a complete-case analysis of 1,794 participants (Figure 1).

### Dependent variable: moderate-to-vigorous physical activity

The outcome variable was the prevalence of meeting the recommended MVPA level. The Japanese version of the WHO Health Behaviour in School-aged Children, a self-report questionnaire developed in a previous study, was used to evaluate MVPA levels.^19^ A validation study indicated that the MVPA results of this questionnaire are significantly and positively correlated with accelerometer data (ρ = 0.339). The questionnaire had the following question: “Over the past 7 days, on how many days were you physically active for at least 60 minutes?” Each student responded with the number of days (0–7 days).

### Independent variables

School grade (fourth, fifth, or sixth grade at PS and first, second, or third grade at JHS) and gender (boy or girl) were evaluated using the self-report questionnaire. Body weight status was calculated from objective measurements of height and weight by a trained school nurse or schoolteacher. Body weight status was classified as thin, normal, or overweight/obese based on the Japanese cut-offs listed in the Health Checkup Manual for School Children, which has been provided by the Ministry of Education, Culture, Sports, Science and Technology, Japan.^20^ Body weight status was calculated as follows:

Body weight status = [measured body weight (kg) − standard body weight for gender, age, and height (kg)]/standard body weight (kg) × 100 (%)

Standard body weight (kg) = *a* × measured body height (cm) − *b*

*a* and *b* are gender- and age-specific scores.

The cut-offs are as follows: overweight/obese: ≥+20%, normal: −20% to +20%, and thinness: ≤−20%.

To measure ST, we employed a previously developed questionnaire to assess children’s ST.^21^ ST (hours per day) on weekdays and weekends was measured for eight domains: (1) television, (2) video or DVD, (3) home console, (4) handheld game console, (5) PC, (6) cellphone, (7) smartphone, and (8) portable music player. For each domain, participants indicated their ST on a six-point Likert scale as follows: “never,” “less than 1 hour,” “1 hour to less than 2 hours,” “2 hours to less than 3 hours,” “3 hours to less than 4 hours,” and “more than 4 hours.” To check the test-retest reliability of the ST questionnaire, 89 students (50 boys and 39 girls) in grades 4–6 (mean age 10.3; standard deviation, 0.8 years) answered the questionnaire (their data were not included in the current study). The test-retest reliability over 7 days for each domain indicated moderate to substantial agreement (weighted kappa = 0.44–0.73). A validation test was not conducted for these items. The total ST was calculated using the total time of domains 1–8 on weekdays and weekends. As a cut-off point, we used less than 2 hours/day based on the guidelines by the American Academy of Pediatrics and the Japan Pediatric Association.^22^^,^^23^

The consumption of breakfast was reported to be associated with PA in previous studies.^24^^,^^25^ We therefore evaluated participants’ frequency of eating breakfast in a week on a five-point Likert scale (every day, 5–6 days, 3–4 days, 1–2 days, or never). Assessment of the test-retest reliability for this item over 7 days indicated substantial agreement (weighted kappa = 0.65). We defined “skipping breakfast” as missing at least one breakfast in a week.

We examined preference for PA as a psychological correlate in this study because it has been shown to be positively associated with PA.^12^^,^^16^ Preference for PA among Japanese children was previously evaluated with a single question asking whether children preferred to engage in PA during school recess.^26^ We evaluated preference for PA using an original item that simply asked, “Do you like physical activity?”. The participants responded on a 4-point Likert scale (yes, somewhat yes, somewhat no, no). For analysis, we dichotomized answers as “like” (yes or somewhat yes) or “dislike” (no or somewhat no).

Population density was calculated by dividing the number of residents by habitable land area in each town; we obtained data from the national population census data from 2015.^27^ Land area figures were constructed from 2014 data obtained from the National Land Numerical Information of the Ministry of Land, Infrastructure, Transport and Tourism of Japan. We calculated the habitable land area by subtracting the non-residential land area (lakes, rivers, and forests) from the total area in each town. Geographic information system was used to calculate the habitable land area (ArcGIS PRO 2.2, ESRI Corporation, Redlands, CA, USA). The population density of Unnan City was 361.7 persons/km^2^ based on the national census. The population densities of the six towns that make up the city were divided into three categories: low-population-density area, 182.3–281.7 persons/km^2^; medium-population-density area, 419.4–483.5 persons/km^2^; and high-population-density area, 532.1–579.6 persons/km^2^.

### Statistical analysis

We first described the participant characteristics. Descriptive statistics were generated to describe the prevalence of meeting recommended MVPA level (and its 95% confidence interval [CI]) according to participants’ school grade and gender.

We then conducted univariate and multivariate Poisson regression analyses to estimate the prevalence ratios (PR) and 95% CIs for having recommended MVPA level (treated as a binary outcome).^28^^,^^29^ We defined the outcome using two separate cut-off points (7 days/week or ≥5 days/week) based on reference values of MVPA of at least 60 minutes/day in accordance with previous studies.^5^^,^^7^^,^^30^^,^^31^ The independent variables (treated as categorical variables) included school grade, gender, body weight status, consumption of breakfast, preference for PA, and population density. To consider the combination of individual and environmental variables, subgroup analyses stratified by gender were used to examine recommended MVPA level with independent variables. None of the variables had correlations of sufficient strength to indicate multicollinearity (*r* < 0.12, data not shown). All statistical analyses were carried out using IBM SPSS Statistics 24.0 for Windows (IBM Corp., Armonk, NY, USA). For all analyses, *P*-values of less than 0.05 were considered statistically significant.

## RESULTS

Table 1 shows the characteristics of total participants by gender. Of the 1,794 participants, a similar proportion of boys and girls participated (949 [52.9%] boys, 845 [47.1%] girls).

**Table 1.** Participants’ characteristics

Variables	Total, *n* = 1,794	Boys, *n* = 949	Girls, *n* = 845
Grade			
Primary school			
4th grades (9–10 years old), *n* (%)	307 (17.1)	163 (17.2)	144 (17.0)
5th grades (10–11 years old), *n* (%)	285 (15.9)	146 (15.4)	139 (16.4)
6th grades (11–12 years old), *n* (%)	282 (15.7)	142 (15.0)	140 (16.6)
Junior high school			
1st grades (12–13 years old), *n* (%)	291 (16.2)	150 (15.8)	141 (16.7)
2nd grades (13–14 years old), *n* (%)	323 (18.0)	183 (19.3)	140 (16.6)
3rd grades (14–15 years old), *n* (%)	306 (17.1)	165 (17.4)	141 (16.7)
Body weight status			
Thin, *n* (%)	28 (1.6)	11 (1.2)	17 (2.0)
Normal, *n* (%)	1,664 (92.8)	886 (93.4)	778 (92.1)
Overweight/obese, *n* (%)	102 (5.7)	52 (5.5)	50 (5.9)
Screen time			
≥2 hours/day, *n* (%)	1,742 (97.1)	925 (97.5)	817 (96.7)
<2 hours/day, *n* (%)	52 (2.9)	24 (2.5)	28 (3.3)
Consumption of breakfast			
Skipping, *n* (%)	165 (9.2)	94 (9.9)	71 (8.4)
Every day, *n* (%)	1,629 (90.8)	855 (90.1)	774 (91.6)
Preference for physical activity			
Dislike, *n* (%)	239 (13.3)	91 (9.6)	148 (17.5)
Like, *n* (%)	1,555 (86.7)	858 (90.4)	697 (82.5)
Population density			
High area, *n* (%)	679 (37.8)	358 (37.7)	321 (38.0)
Medium area, *n* (%)	919 (51.2)	495 (52.2)	424 (50.2)
Low area, *n* (%)	196 (10.9)	96 (10.1)	100 (11.8)

**Figure 2.**
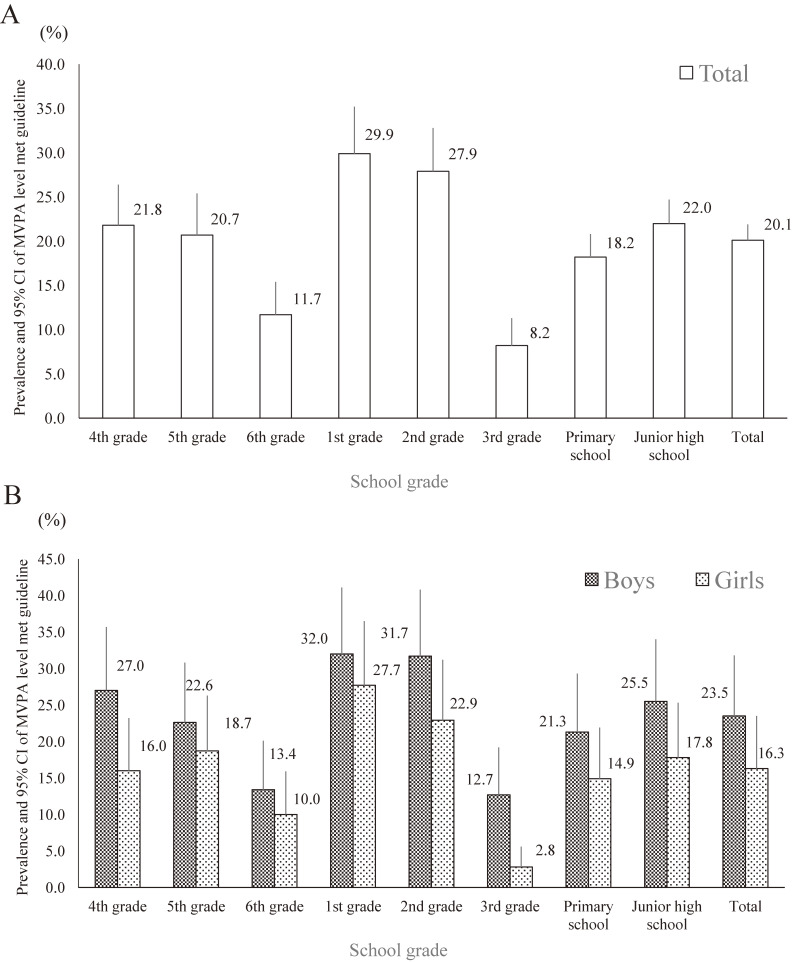
Prevalence and 95% confidence intervals (CIs) of engaging in recommended level of moderate-to-vigorous physical activity in the total (A) sample and differences between boys and girls (B). Primary school grade is 4th grade (9–10 years old), 5th grade (10–11 years old), and 6th grade (11–12 years old). Junior high school grade is 1st grade (12–13 years old), 2nd grade (13–14 years old), and 3rd grade (14–15 years old).

Figure 2 and eTable 1 display the prevalence of meeting recommended MVPA level (≥60 minutes/day on 7 days/week) among Japanese children and adolescents by grade and gender. Overall, 20.1% of participants met recommended level.

Table 2 shows the results of the Poisson regression exploring the associations of meeting recommended MVPA level with the independent variables. When interpreting the recommended level as ≥60 minutes/day for 7 days, meeting this recommendation was significantly associated with being in sixth grade of PS (PR 0.57; 95% CI, 0.39–0.84) and first (PR 1.52; 95% CI, 1.16–1.99), second (PR 1.45; 95% CI, 1.10–1.90), and third grade of JHS (PR 0.40; 95% CI, 0.26–0.62) (vs fourth grade of PS). It was also associated with being a boy (PR 1.33; 95% CI, 1.12–1.59) (vs girl), liking PA (PR 3.72; 95% CI, 2.22–6.22) (vs dislike), and living in a medium-population-density (PR 0.73; 95% CI, 0.61–0.88) or low-population-density area (PR 0.67; 95% CI, 0.48–0.94) (vs high-population-density area) in the multivariate analysis.

**Table 2.** Correlates for moderate-to-vigorous physical activity among Japanese children and adolescents (*n* = 1,794)

		MVPA level, ≥60 minutes/day on 7 days/week			MVPA level, ≥60 minutes/day on ≥5 days/week		

		*n* (%)	univariate	multivariate	*n* (%)	univariate	multivariate
	
			PR (95% CI)	PR (95% CI)		PR (95% CI)	PR (95% CI)
School grade	4th grades	67 (21.8)	1.0 (reference)	1.0 (reference)	157 (48.9)	1.0 (reference)	1.0 (reference)
	5th grades	59 (20.7)	0.95 (0.70–1.29)	1.01 (0.75–1.37)	121 (42.5)	0.87 (0.73–1.04)	0.89 (0.75–1.07)
	6th grades	33 (11.7)	**0.54 (0.37–0.79)**	**0.57 (0.39–0.84)**	97 (34.4)	**0.70 (0.58–0.86)**	**0.73 (0.60–0.89)**
	1st grades	87 (29.9)	**1.37 (1.04–1.80)**	**1.52 (1.16–1.99)**	181 (62.2)	**1.27 (1.10–1.47)**	**1.34 (1.16–1.54)**
	2nd grades	90 (27.9)	1.28 (0.97–1.68)	**1.45 (1.10–1.90)**	174 (53.9)	1.10 (0.95–1.28)	**1.17 (1.01–1.36)**
	3rd grades	25 (8.2)	**0.37 (0.24–0.58)**	**0.40 (0.26–0.62)**	66 (21.6)	**0.44 (0.35–0.56)**	**0.45 (0.36–0.58)**

Gender	Girls	138 (16.3)	1.0 (reference)	1.0 (reference)	339 (40.1)	1.0 (reference)	1.0 (reference)
	Boys	223 (23.5)	**1.44 (1.19–1.74)**	**1.33 (1.12–1.59)**	450 (47.4)	**1.18 (1.06–1.31)**	**1.13 (1.02–1.25)**

Body weight status	Thin	2 (7.1)	0.34 (0.09–1.31)	0.34 (0.09–1.27)	10 (35.7)	0.81 (0.49–1.33)	0.76 (0.47–1.20)
	Normal	346 (20.8)	1.0 (reference)	1.0 (reference)	737 (44.4)	1.0 (reference)	1.0 (reference)
	Overweight/obese	13 (12.7)	0.61 (0.37–1.03)	0.75 (0.45–1.24)	41 (40.2)	0.91 (0.71–1.16)	0.99 (0.78–1.26)

Screen time	≥2 hours/day	348 (20.0)	1.0 (reference)	1.0 (reference)	766 (44.0)	1.0 (reference)	1.0 (reference)
	<2 hours/day	13 (25.0)	1.25 (0.77–2.02)	1.29 (0.83–1.99)	23 (44.2)	1.01 (0.74–1.37)	1.00 (0.74–1.34)

Consumption of breakfast	Skipping	21 (12.7)	1.0 (reference)	1.0 (reference)	60 (36.4)	1.0 (reference)	1.0 (reference)
	Every day	340 (20.9)	**1.64 (1.09–2.47)**	1.47 (0.98–2.19)	729 (44.8)	1.23 (1.00–1.52)	1.15 (0.94–1.41)

Preference for physical activity	Dislike	14 (5.9)	1.0 (reference)	1.0 (reference)	63 (26.4)	1.0 (reference)	1.0 (reference)
	Like	347 (22.3)	**3.81 (2.27–6.39)**	**3.72 (2.22–6.22)**	726 (46.7)	**1.77 (1.42–2.20)**	**1.79 (1.45–2.22)**

Population density	High area	165 (24.3)	1.0 (reference)	1.0 (reference)	313 (46.1)	1.0 (reference)	1.0 (reference)
	Medium area	166 (18.1)	**0.74 (0.61–0.90)**	**0.73 (0.61–0.88)**	406 (44.2)	0.96 (0.86–1.07)	0.94 (0.85–1.05)
	Low area	30 (15.3)	**0.63 (0.44–0.90)**	**0.67 (0.48–0.94)**	70 (35.7)	**0.76 (0.63–0.95)**	**0.79 (0.65–0.96)**

CI, confidence interval; MVPA, moderate-to-vigorous physical activity; PR, prevalence ratio.

School grade, Primary school grade is 4th grade (9–10 years old), 5th grade (10–11 years old), and 6th grade (11–12 years old). Junior high school grade is 1st grade (12–13 years old), 2nd grade (13–14 years old), and 3rd grade (14–15 years old).

When interpreting recommended MVPA level as ≥60 minutes/day for at least 5 days/week, meeting the level was associated with being in sixth grade of PS (PR 0.73; 95% CI, 0.60–0.89) and first (PR 1.34; 95% CI, 1.16–1.54), second (PR 1.17; 95% CI, 1.01–1.36), and third grade of JHS (PR 0.45; 95% CI, 0.36–0.58) (vs fourth grade of PS). It was also associated with being a boy (PR 1.13; 95% CI, 1.02–1.25) (vs girl), liking PA (PR 1.79; 95% CI, 1.45–2.22) (vs dislike), and living in a low-population-density (PR 0.79; 95% CI, 0.65–0.96) (vs high-population-density area) area in the multivariate analysis.

In eTable 2, the subgroup analysis for boys revealed that meeting recommended MVPA level as ≥60 minutes/day for 7 days was significantly associated with being in sixth grade of PS (PR 0.52; 95% CI, 0.32–0.84) and third grade of JHS (PR 0.50; 95% CI, 0.31–0.81), liking PA (PR 3.91; 95% CI, 1.83–8.38), and live in a medium-population-density area (PR 0.73; 95% CI, 0.58–0.92). For girls, factors associated with higher MVPA level were being in first (PR 2.02; 95% CI, 1.29–3.16), second (PR 1.67; 95% CI, 1.05–2.67), and third grade of JHS (PR 0.19; 95% CI, 0.07–0.54), liking PA (PR 3.53; 95% CI, 1.76–7.11), and living in a medium-population-density (PR 0.74; 95% CI, 0.55–0.99) or low-population-density area (PR 0.49; 95% CI, 0.27–0.90).

## DISCUSSION

This is the first study to describe the prevalence of meeting WHO-recommended MVPA level using census data of children and adolescents living in Unnan City, Japan. We also examined the numerous correlates of this prevalence. About 20% of the Japanese children and adolescents met recommended MVPA level, which was associated with grade, gender, preference for PA, and population density. Our findings underscore the importance of promoting MVPA among Japanese children and adolescents. Based on attributes of students not meeting recommended MVPA level in the Japanese rural area, measures focusing on increasing PA among students in the last grade of schools or in low-population-density areas may be necessary. For example, there is a need for multidisciplinary cooperation of stakeholders, such as teachers, parents, and education policymakers.

### Prevalence of moderate-to-vigorous physical activity that meets the WHO recommendation

We found that 20.1% of Japanese children and adolescents aged 9–15 years achieved recommended MVPA level. The prevalence of meeting recommended MVPA level in Japanese children and adolescents is low, a finding which is consistent with the international trend.^30^ This is similar to the prevalence found among adolescents aged 13–15 years in 105 countries (19.7%) assessed by HBSC and GSHS.^30^ Even if the results are limited to the same age group (JHS aged 13–15 years) in our study, the prevalence was low (22.0%). The GSHS also found that 19.3% of children and adolescents aged 11–17 years met the recommendation globally.^6^ Among Asian countries, according to the WHO Global Health Observatory data (from the GSHS), Southeast Asian countries had a distinctly higher prevalence of meeting recommended MVPA level (26.6%) than did Japan.^32^ For example, although the age classification was different, Amornsriwatanakul et al conducted a survey using a validated questionnaire among 13,225 Thai children and youth aged 6–17 years,^33^ and 23.4% met recommended MVPA level (vs 20.1% in Japan). As mentioned above, to the best of our knowledge, no study has yet examined the prevalence of meeting recommended MVPA level at the population level in Japan. Therefore, our findings are important for public health, because we analyzed participants from all schools in an entire city, thus decreasing sampling bias and obtaining results more reflective of reality.

### Correlates with moderate-to-vigorous physical activity level

Being a boy was positively correlated with meeting recommended MVPA level. This finding accords with international trends.^15^ Being in the final year of a particular school (sixth grade of PS and third grade of JHS) was negatively associated with meeting recommended level, consistent with the findings of Fukushima et al, who reported that students in their final years of PS or JHS had the lowest step counts using a pedometer in the Tokyo metropolitan area.^10^ The lower prevalence might be attributed to the fact that Japanese students stop participating in organized sports between July and March to prepare for examinations and graduation. Childhood is a key developmental period when health-related behaviors are established and can be carried into adulthood. According to Telama’s review,^3^ tracking coefficients from childhood to adulthood ranged between 0.07 and 0.66. Moreover, participants who were consistently inactive were more likely to become obese in young adulthood, compared to consistently active participants (adjusted odds ratio, 3.79).^4^ It may therefore be necessary to take further measures to improve MVPA for students in their final year of PS and JHS in Japan.

Liking PA was the strongest positive correlate of meeting the MVPA recommendations, regardless of whether we set the cut-off as 7 or ≥5 days/week. These findings support previous reviews showing that liking PA is positively correlated with actual participation in PA among children and adolescents.^12^^,^^16^ Qualitative studies have explored the reasons that adolescents participate in PA, including sports. The most common reasons appear to be skill development, challenges that PA can present, consequent improvement in fitness and physical competence, and opportunity to gain social acceptance and support as well as have fun.^34^^,^^35^ Thus, fun or enjoyment was a common reason for participation in PA. One approach to exploring this association is motivation to engage in PA. According to self-determination theory, motivation has intrinsic and extrinsic aspects.^36^ Intrinsic motivation refers to engaging in a behavior that is inherently satisfying, interesting, or enjoyable. Intrinsic motivation is an important factor for remaining physically active.^37^ Owen’s review showed that intrinsic motivation is associated with engaging in PA among children and adolescents.^38^ In our study, liking PA was the independent variable with the strongest association with MVPA. However, although the percentage of students liking PA was high (86.7%), prevalence of meeting recommended MVPA level was low (20.1%). There may be potential mediators, such as self-efficacy, between liking PA and engaging in MVPA.^39^ Our findings suggest that to promote MVPA, not only should parents or teachers aim to present PA as at least a fun activity for children, more research should be conducted to identify potential effects of other variables.

We also found a positive relationship between population density and MVPA among Japanese children and adolescents. This positive relationship was observed in both boys and girls. In a systematic review among East Asian adolescents, there are few studies on the environmental correlates of participation in MVPA.^40^ This review showed inconsistencies in findings regarding the association between residential density and PA. In Japan, Sato et al showed a reverse U-shaped relationship between population density and step count (the lowest and highest quintiles of population density showed a decline in step count) using a pedometer from the 2011 Tokyo Metropolitan Survey, which assessed youth aged 6–15 years.^41^ Our findings that lower population density was associated with a lower prevalence of meeting recommended MVPA level compared with a high population density partly supported their results. In our study area, the mean population density was 361.7 persons/km^2^, which is lower than the mean of the least dense area of Tokyo (532 persons/km^2^). This lower population density might be a less favorable environment for MVPA. Moreover, Sato et al showed that population density had a comparatively stronger influence on out-of-school step count and a weaker influence on in-school step count. We speculate that out-of-school PA is important for the total amount of PA among children and adolescents, because the number of sports clubs in Shimane Prefecture (including in our study area) decreased from 339 in 2002 to 289 in 2014, and the average number of participating members decreased from 26.0 people/club to 21.5 people/club.^42^ With the decrease in the number of sports clubs, opportunities for participation in out-of-school sports activities might have decreased. Moreover, the presence of school buses might also decrease transportation using walking or cycling in areas with low-population-density. In Japan, most students (86% according to a previous study^9^) walk or cycle to commute from home to school. However, in areas with low densities (eg, rural communities), it can be difficult to commute via these methods, forcing students to use the school bus. D’Haese et al’s systematic review reported that walking to school was positively associated with population density.^43^ Nevertheless, there is a lack of evidence on this issue in Asian countries. Our study examined overall level of MVPA, presumably including activities in schools and clubs, but did not assess domain-specific activities (ie, physical education, participating in organized sports, or transportation). Future research should enhance the understanding of mechanisms underlying the association between PA (both in and out of school) and population density.

This study has several potential limitations. First, the cross-sectional design cannot explain the causal relationship between the MVPA and its correlates. Second, although we recruited participants from all schools of city, the generalizability of the findings is limited because this study was performed in a single rural Japanese city. Additionally, there might have been a bias due to missing data through non-participation or non-response to the questionnaires. Students with missing data (*n* = 104) were slightly more active than students of complete case (21.2% vs 20.1%). When examining this by school grade, the similar but greater tendency was observed in the fifth grade (24.0% vs 20.7%), which had the highest rate of missing data. Therefore, the missing data might lead to underestimating the prevalence and correlates of recommended MVPA level. A future study with a nationally representative sample that includes rural and urban areas is needed. Third, despite the use of a validated questionnaire to measure MVPA, recall bias might have influenced results. Subjective measurements tend to overestimate PA compared with objective measurements.^44^^,^^45^ Furthermore, although the item for measuring preference for PA was developed in a previous study in Japan, its validity and reliability are unknown.^26^ A validation test was not conducted for assessing other independent variables using a questionnaire. Therefore, the results are subject to measurement error. Finally, we could not account for the effects of unmeasured factors, which might have influenced the confounders of MVPA.^12^^–^^17^

### Conclusions

This study explored the prevalence of meeting WHO-recommended MVPA level and its correlates among Japanese children and adolescents. About 20% students engaged in the recommended level, and prevalence was associated with grade, gender, preference for PA, and population density.

## APPENDIX A. SUPPLEMENTARY DATA

eTable 1. Prevalence of moderate-to-vigorous physical activity levels among Japanese children and adolescents

eTable 2. Gender-stratified correlates for moderate-to-vigorous physical activity among Japanese children and adolescents

## References

[1] Janssen I, Leblanc AG Systematic review of the health benefits of physical activity and fitness in school-aged children and youth. Int J Behav Nutr Phys Act. 2010;7:40. 10.1186/1479-5868-7-40 20459784 PMC2885312

[2] Biddle SJ, Asare M Physical activity and mental health in children and adolescents: a review of reviews. Br J Sports Med. 2011;45:886–895. 10.1136/bjsports-2011-090185 21807669

[3] Telama R Tracking of physical activity from childhood to adulthood: a review. Obes Facts. 2009;2:187–195. 10.1159/000222244 20054224 PMC6516203

[4] Kwon S, Janz KF, Letuchy EM, Burns TL, Levy SM Active lifestyle in childhood and adolescence prevents obesity development in young adulthood. Obesity (Silver Spring). 2015;23:2462–2469. 10.1002/oby.21262 26538514 PMC4701632

[5] World Health Organization. Global recommendations on physical activity for health. Geneva: World Health Organization; 2010. Available from: https://www.who.int/dietphysicalactivity/factsheet_recommendations/en/ [Accessed 2019. 2. 2.]. 26180873

[6] World Health Organization. Global action plan on physical activity 2018–2030: more active people for a healthier world. Geneva: World Health Organization; 2018. Available from: https://www.who.int/ncds/prevention/physical-activity/global-action-plan-2018-2030/en/ [Accessed 2019. 2. 2.].

[7] Peltzer K, Pengpid S Leisure time physical inactivity and sedentary behaviour and lifestyle correlates among students aged 13–15 in the Association of Southeast Asian Nations (ASEAN) Member States, 2007–2013. Int J Environ Res Public Health. 2016;13:217. 10.3390/ijerph13020217 26891312 PMC4772237

[8] Sallis JF, Bull F, Guthold R, ; Lancet Physical Activity Series 2 Executive Committee Progress in physical activity over the Olympic quadrennium. Lancet. 2016;388:1325–1336. 10.1016/S0140-6736(16)30581-5 27475270

[9] Tanaka C, Tanaka S, Inoue S, Results from the Japan’s 2018 report card on physical activity for children and youth. J Exerc Sci Fit. 2019;17:20–25. 10.1016/j.jesf.2018.10.001 30662510 PMC6323183

[10] Fukushima N, Inoue S, Hikihara Y, Pedometer-determined physical activity among youth in the Tokyo Metropolitan area: a cross-sectional study. BMC Public Health. 2016;16:1104. 10.1186/s12889-016-3775-5 27769277 PMC5073463

[11] Yamakita M, Sato M, Suzuki K, Ando D, Yamagata Z Sex differences in birth weight and physical activity in Japanese schoolchildren. J Epidemiol. 2018;28:331–335. 10.2188/jea.JE20170078 29479002 PMC6004363

[12] Sallis JF, Prochaska JJ, Taylor WC A review of correlates of physical activity of children and adolescents. Med Sci Sports Exerc. 2000;32:963–975. 10.1097/00005768-200005000-00014 10795788

[13] Biddle SJ, Atkin AJ, Cavill N, Foster C Correlates of physical activity in youth: a review of quantitative systematic reviews. Int Rev Sport Exerc Psychol. 2011;4:25–49. 10.1080/1750984X.2010.548528

[14] Craggs C, Corder K, van Sluijs EM, Griffin SJ Determinants of change in physical activity in children and adolescents: a systematic review. Am J Prev Med. 2011;40:645–658. 10.1016/j.amepre.2011.02.025 21565658 PMC3100507

[15] Bauman AE, Reis RS, Sallis JF, Wells JC, Loos RJ, Martin BW; Lancet Physical Activity Series Working Group Correlates of physical activity: why are some people physically active and others not? Lancet. 2012;380:258–271. 10.1016/S0140-6736(12)60735-1 22818938

[16] Brown H, Hume C, Pearson N, Salmon J A systematic review of intervention effects on potential mediators of children’s physical activity. BMC Public Health. 2013;13:165. 10.1186/1471-2458-13-165 23433143 PMC3585884

[17] McGrath LJ, Hopkins WG, Hinckson EA Associations of objectively measured built-environment attributes with youth moderate-vigorous physical activity: a systematic review and meta-analysis. Sports Med. 2015;45:841–865. 10.1007/s40279-015-0301-3 25618013

[18] Sallis JF, Owen N, Fisher E. Ecological models of health behavior. In: Glanz K, Rimer BK, Viswanath K, eds. *Health behavior and health education: theory, research and practice*. San Francisco, CA, US: Jossey-Bass; 2008:465–485.

[19] Tanaka C, Kyan A, Takakura M, The validity of the Japanese version of physical activity questions in the WHO Health Behaviour in School-aged Children (HBSC) survey. Jpn Assoc Exerc Epidemiol. 2018;19:93–101.

[20] Ministry of Education, Culture, Sports, Science and Technology. Health Checkup Manual for School Children (2015 Revision). Japan Society of School Health; 2015. Available from: https://www.gakkohoken.jp/book/ebook/ebook_H270030/index_h5.html [Accessed 2019. 2. 2.] (in Japanese).

[21] Yamada M, Sekine M, Tatsuse T Parental internet use and lifestyle factors as correlates of prolonged screen time of children in Japan: results From the Super Shokuiku School Project. J Epidemiol. 2018;28:407–413. 10.2188/jea.JE20170100 29576604 PMC6143380

[22] American Academy of Pediatrics, Committee on Public Education American Academy of Pediatrics: children, adolescents, and television. Pediatrics. 2001;107:423–426. 10.1542/peds.107.2.423 11158483

[23] The Japan Pediatric Association. Proposal for children’s media use; 2004. Available from: http://www.jpa-web.org/dcms_media/other/ktmedia_teigenzenbun.pdf [Accessed 2019. 2. 2.] (in Japanese).

[24] Tin SP, Ho SY, Mak KH, Wan KL, Lam TH Lifestyle and socioeconomic correlates of breakfast skipping in Hong Kong primary 4 schoolchildren. Prev Med. 2011;52:250–253. 10.1016/j.ypmed.2010.12.012 21215276

[25] Corder K, van Sluijs EM, Steele RM, Breakfast consumption and physical activity in British adolescents. Br J Nutr. 2011;105:316–321. 10.1017/S0007114510003272 20807464 PMC3361684

[26] Noda K, Shikano A, Noi S Factors affecting motivation for independent physical activity during elementary school recess times in 3rd and 6th graders. Japan J Hum Growth Dev Res. 2017;2017:1–16 (in Japanese) 10.5332/hatsuhatsu.2017.75_1

[27] Bureau of the Census, Ministry of Internal Affairs and Communications. Population census. 2015. Available from: https://www.stat.go.jp/data/kokusei/2015/kekka.html [Accessed 2019. 2. 2.] (in Japanese).

[28] Zhang J, Yu KF What’s the relative risk? A method of correcting the odds ratio in cohort studies of common outcomes. JAMA. 1998;280:1690–1691. 10.1001/jama.280.19.1690 9832001

[29] Barros AJ, Hirakata VN Alternatives for logistic regression in cross-sectional studies: an empirical comparison of models that directly estimate the prevalence ratio. BMC Med Res Methodol. 2003;3:21. 10.1186/1471-2288-3-21 14567763 PMC521200

[30] Hallal PC, Andersen LB, Bull FC, Guthold R, Haskell W, Ekelund U; Lancet Physical Activity Series Working Group Global physical activity levels: surveillance progress, pitfalls, and prospects. Lancet. 2012;380:247–257. 10.1016/S0140-6736(12)60646-1 22818937

[31] Janssen I, Katzmarzyk PT, Boyce WF, ; Health Behaviour in School-Aged Children Obesity Working Group Comparison of overweight and obesity prevalence in school-aged youth from 34 countries and their relationships with physical activity and dietary patterns. Obes Rev. 2005;6:123–132. 10.1111/j.1467-789X.2005.00176.x 15836463

[32] World Health Organization. *Global Health Observatory data repository: Prevalence of insufficient physical activity among school going adolescents*. Geneva: World Health Organization; 2010.

[33] Amornsriwatanakul A, Lester L, Bull FC, Rosenberg M Are Thai children and youth sufficiently active? prevalence and correlates of physical activity from a nationally representative cross-sectional study. Int J Behav Nutr Phys Act. 2017;14:72. 10.1186/s12966-017-0529-4 28558779 PMC5450254

[34] Frederick CM, Ryan RM Differences in motivation for sport and exercise and their relations with participation and mental health. J Sport Behav. 1993;16:124–126.

[35] Weiss MR Motivating kids in physical activity. President’s Council on Physical Fitness and Sports Research Digest. 2000;3(11):1–10. PMC3022443 21253445

[36] Deci E, Ryan RM. *Intrinsic motivation and self-determination in human behavior*. New York: Springer; 1985.

[37] Ryan RM, Deci EL. Active human nature: Self-determination theory and the promotion and maintenance of sport, exercise, and health. In: Hagger MS, Chatzisarantis NLD, eds. *Intrinsic motivation and self-determination in exercise and sport*. Champaign IL: Human Kinetics; 2007:1–19.

[38] B Owen K, Smith J, Lubans DR, Ng JY, Lonsdale C Self-determined motivation and physical activity in children and adolescents: a systematic review and meta-analysis. Prev Med. 2014;67:270–279. 10.1016/j.ypmed.2014.07.033 25073077

[39] Wang JJ, Baranowski T, Lau PWC, Psychological correlates of self-reported and objectively measured physical activity among Chinese children—Psychological correlates of PA. Int J Environ Res Public Health. 2016;13:1006 10.3390/ijerph13101006 PMC5086745 27754396

[40] Lee LL, Kuo YL, Chan ESY The association between built environment attributes and physical activity in East Asian Adolescents: a systematic review. Asia Pac J Public Health. 2016;28:206–218. 10.1177/1010539516628174 26969634

[41] Sato H, Inoue S, Fukushima N, Lower youth steps/day values observed at both high and low population density areas: a cross-sectional study in metropolitan Tokyo. BMC Public Health. 2018;18:1132. 10.1186/s12889-018-6028-y 30236088 PMC6149053

[42] Japan Sports Association, Sasakawa Sports Foundation. Report: the change in the numbers of Junior Sports Clubs and its members (2002–2014). 2016. Available from: http://www.ssf.or.jp/Portals/0/resources/research/report/pdf/report_201610_all.pdf. [Accessed 2019. 2. 2.] (in Japanese).

[43] D’Haese S, Vanwolleghem G, Hinckson E, Cross-continental comparison of the association between the physical environment and active transportation in children: a systematic review. Int J Behav Nutr Phys Act. 2015;12:145. 10.1186/s12966-015-0308-z 26610344 PMC4660808

[44] Adamo KB, Prince SA, Tricco AC, Connor-Gorber S, Tremblay M A comparison of indirect versus direct measures for assessing physical activity in the pediatric population: a systematic review. Int J Pediatr Obes. 2009;4:2–27. 10.1080/17477160802315010 18720173

[45] Ekelund U, Tomkinson G, Armstrong N What proportion of youth are physically active? Measurement issues, levels and recent time trends. Br J Sports Med. 2011;45:859–865. 10.1136/bjsports-2011-090190 21836170

