# Supplementary material for: Prevalence and Correlates of Physical Activity Among Children and Adolescents: A Cross-Sectional Population-Based Study of a Rural City in Japan
Source: J Epidemiol. 2020 Sep 5;30(9):404–11. doi: 10.2188/jea.JE20190047 (PMC7429150; doi:10.2188/jea.JE20190047)
Supplement: Supplementary file 1 [file je-30-404-s001.pdf]

eTable 1. Prevalence of moderate-to-vigorous physical activity levels among Japanese children and adolescents

Variables	Total, n=1,794		Boys, n=949		Girls, n=845	
	n (%)	95% CI	n (%)	95% CI	n (%)	95% CI
MVPA level, ≥60 minutes/day on 7 days/week						
Primary school	159 (18.2)	(15.6 – 20.7)	96 (21.3)	(17.5 – 25.1)	63 (14.9)	(11.5 – 18.3)
4th grade (9–10 years old)	67 (21.8)	(17.2 – 26.4)	44 (27.0)	(20.2 – 33.8)	23 (16.0)	(10.0 – 22.0)
5th grade (10–11 years old)	59 (20.7)	(16.0 – 25.4)	33 (22.6)	(15.8 – 29.4)	26 (18.7)	(12.2 – 25.2)
6th grade (11–12 years old)	33 (11.7)	(8.0 – 15.5)	19 (13.4)	(7.8 – 19.0)	14 (10.0)	(5.0 – 15.0)
Junior high school	202 (22.0)	(19.3 – 24.6)	127 (25.5)	(21.7 – 29.3)	75 (17.8)	(14.1 – 21.4)
1st grade (12–13 years old)	87 (29.9)	(24.6 – 35.2)	48 (32.0)	(24.5 – 39.5)	39 (27.7)	(20.3 – 35.0)
2nd grade (13–14 years old)	90 (27.9)	(23.0 – 32.8)	58 (31.7)	(25.0 – 38.4)	32 (22.9)	(15.9 – 29.8)
3rd grade (14–15 years old)	25 (8.2)	(5.1 – 11.2)	21 (12.7)	(7.6 – 17.8)	4 (2.8)	(0.1 – 5.6)
Total	361 (20.1)	(18.3 – 22.0)	223 (23.5)	(20.8 – 26.2)	138 (16.3)	(13.8 – 18.8)
MVPA level, ≥60 minutes/day on ≥5 days/week						
Primary school	368 (42.1)	(38.8 – 45.4)	192 (42.6)	(38.0 – 47.1)	176 (41.6)	(36.9 – 46.3)
4th grade (9–10 years old)	150 (48.9)	(43.3 – 54.5)	82 (50.3)	(42.6 – 58.0)	68 (47.2)	(39.1 – 55.4)
5th grade (10–11 years old)	121 (42.5)	(36.7 – 48.2)	63 (43.2)	(35.1 – 51.2)	58 (41.7)	(33.5 – 49.9)
6th grade (11–12 years old)	97 (34.4)	(28.9 – 39.9)	47 (33.1)	(25.4 – 40.8)	50 (35.7)	(27.8 – 43.7)
Junior high school	421 (45.8)	(42.5 – 49.0)	258 (51.8)	(47.4 – 56.2)	163 (38.6)	(34.0 – 43.8)
1st grade (12–13 years old)	181 (62.2)	(56.6 – 67.8)	107 (71.3)	(64.1 – 78.6)	74 (52.5)	(44.2 – 60.7)
2nd grade (13–14 years old)	174 (53.9)	(48.4 – 59.3)	105 (57.4)	(50.2 – 64.5)	69 (49.3)	(41.0 – 57.6)
3rd grade (14–15 years old)	66 (21.6)	(17.0 – 26.2)	46 (27.9)	(21.0 – 34.7)	20 (14.2)	(8.4 – 19.9)
Total	789 (44.0)	(41.7 – 46.3)	450 (47.4)	(44.2 – 50.6)	339 (40.1)	(36.8 – 43.4)

CI, confidence interval; MVPA, moderate-to-vigorous physical activity.

eTable 2. Gender-stratified correlates for moderate-to-vigorous physical activity among Japanese children and adolescents

		Boys (n=949)		Girls (n=845)	
		MVPA, ≥60 min on 7 days/week	MVPA, ≥60 min on ≥5 days/week	MVPA, ≥60 min on 7 days/week	MVPA, ≥60 min on ≥5 days/week
		PR (95% CI)	PR (95% CI)	PR (95% CI)	PR (95% CI)
School grade	4th grades	1.0 (reference)	1.0 (reference)	1.0 (reference)	1.0 (reference)
	5th grades	0.87 (0.59 – 1.28)	0.87 (0.69 – 1.11)	1.31 (0.79 – 2.16)	0.91 (0.70 – 1.19)
	6th grades	**0.52 (0.32 – 0.84)**	**0.67 (0.51 – 0.89)**	0.70 (0.38 – 1.29)	0.79 (0.59 – 1.05)
	1st grades	1.26 (0.90 – 1.78)	**1.46 (1.22 – 1.75)**	**2.02 (1.29 – 3.16)**	1.18 (0.93 – 1.49)
	2nd grades	1.33 (0.96 – 1.86)	**1.22 (1.01 – 1.49)**	**1.67 (1.05 – 2.67)**	1.10 (0.87 – 1.40)
	3rd grades	**0.50 (0.31 – 0.81)**	**0.58 (0.43 – 0.77)**	**0.19 (0.07 – 0.54)**	**0.30 (0.20 – 0.47)**
Body weight status	Thin	0.00 (0.00 – 0.00)	0.68 (0.29 – 1.58)	0.48 (0.13 – 1.74)	0.82 (0.47 – 1.44)
	Normal	1.0 (reference)	1.0 (reference)	1.0 (reference)	1.0 (reference)
	Overweight/obese	0.78 (0.43 – 1.43)	0.97 (0.72 – 1.31)	0.69 (0.28 – 1.74)	0.97 (0.65 – 1.44)
Screen time	≥2 hours/day	1.0 (reference)	1.0 (reference)	1.0 (reference)	1.0 (reference)
	<2 hours/day	1.54 (0.91 – 2.60)	1.19 (0.85 – 1.66)	1.03 (0.48 – 2.21)	0.78 (0.48 – 1.28)
Consumption of breakfast	Skipping	1.0 (reference)	1.0 (reference)	1.0 (reference)	1.0 (reference)
	Every day	1.29 (0.82 – 2.04)	1.20 (0.92 – 1.57)	2.18 (0.94 – 5.03)	1.11 (0.81 – 1.53)
Preference for physical activity	Dislike	1.0 (reference)	1.0 (reference)	1.0 (reference)	1.0 (reference)
	Like	**3.91 (1.83 – 8.38)**	**1.96 (1.40 – 2.73)**	**3.53 (1.76 – 7.11)**	**1.68 (1.26 – 2.23)**
Population density	High area	1.0 (reference)	1.0 (reference)	1.0 (reference)	1.0 (reference)
	Medium area	**0.73 (0.58 – 0.92)**	0.99 (0.86 – 1.13)	**0.74 (0.55 – 0.99)**	0.88 (0.75 – 1.04)
	Low area	0.80 (0.53 – 1.19)	0.93 (0.73 – 1.19)	**0.49 (0.27 – 0.90)**	**0.64 (0.47 – 0.87)**

CI, confidence interval; MVPA, moderate-to-vigorous physical activity; PR, prevalence ratio.

School grade, Primary school grade is 4th grade (9–10 years old), 5th grade (10–11 years old), and 6th grade (11–12 years old). Junior high school grade is 1st grade (12–13 years old), 2nd grade (13–14 years old), and 3rd grade (14–15 years old).
